# What factors influence clinical decision making for paramedics when attending to paediatric emergencies in the community within one ambulance service trust?

**DOI:** 10.29045/14784726.2021.6.6.1.15

**Published:** 2021-05-01

**Authors:** Jeff Hetherington, Ian Jones

**Affiliations:** Alder Hey Hospital; North West Ambulance Service; Liverpool John Moores University

**Keywords:** clinical decisions, decision making, paediatric, paramedic

## Abstract

**Background:**

Children’s healthcare needs are complex and diverse. Paramedics are expected to respond to a range of emergency calls across the patient demographic spectrum and to make complex clinical decisions, whilst facing growing pressures to seek provisions of care for their patients within the community.

**Aim:**

This study looked to understand the lived experiences of paramedics when attending to paediatric patients, and what factors influenced decision making.

**Methods:**

A qualitative study employing semi-structured interviews, to collect and describe the lived experiences of participants. Participants were paramedics working for an ambulance service responding to calls in the community. Participants varied in experience and registrant level of education. Interview data were transcribed verbatim and analysed utilising inductive thematic analysis.

**Results:**

- Education provoked the most discussion, with a desire for more knowledge and training to improve confidence when attending to patients with low acuity complaints.
- Confidence was found to be intrinsically linked to experience, with clinicians who were more exposed to paediatrics in their professional or personal life displaying more confidence when attending to this patient group.
- Emotion of clinicians and/or families contributes to the clinical decision-making process; coupled with a reliance on clinical guidelines, there is a high probability of a paediatric consultation resulting in conveyance to an emergency department.
- Provision of care was variable geographically, with largely negative experiences observed with attempts for community referrals.

**Conclusions:**

Providing a focus of education more reflective of paramedics’ experiences will address some of the factors discussed by participants. Introducing innovative solutions such as developing guidelines for lower acuity conditions and the introduction of specialist roles could contribute to mitigating the barriers paramedics faced while improving the quality of care provided to paediatric patients. Barriers to confidence existing due to lack of exposure may well still exist, as would options to refer to community services.

## Introduction

The NHS England (2019) ‘long term plan’ recognises that children’s health needs are diverse and complex, with the Royal College of Paediatrics and Child Health (2018) highlighting that children account for 25% of attendances to emergency departments (EDs). Many of these attendances would be suitable to be managed in the community (McHale et al., 2013). The emergency care system is under pressure (NHS England, 2019), requiring differing models of care outside of EDs to be sought. O’Cathain et al. (2018) recognised that discharge at scene is a complex decision for paramedics and is often made in isolation (O’Hara et al., 2014).

In an unpublished internal paper, the North West Ambulance Service acknowledged that total calls to the service for patients under the age of 16 accounted for approximately 9% total call volume. See and treat figures of approximately 13% for this patient group were, however, disproportionately low in comparison with patients over the age of 16 (North West Ambulance Service NHS Trust, 2018).

Houston and Pearson’s (2009) literature review identified a requirement for an up-to-date assessment and evaluation of service provision from the UK ambulance services for children. The evaluation highlighted that training still had little paediatric input. Data surrounding care provided and practitioner confidence were also noted to be lacking.

*Taking healthcare to the patient* (Department of Health, 2005), *Transforming NHS ambulance services* (National Audit Office, 2011) and *Taking healthcare to the patient 2* (Association of Ambulance Chief Executives, 2011) have all contributed to positive transformation of the ambulance service model of care but within them there has been little mention specific to paediatric care.

Fowler et al.’s (2017) scoping review of the literature surrounding paramedic confidence with paediatric patients revealed feelings of low confidence and efficacy. This was underpinned by anxiety and showed correlation with a potential of lower quality care for paediatric patients and reluctance to initiate treatment. The study reviewed 17 articles primarily from the US.

The aim of this study was to understand the lived experiences of paramedics from one ambulance service trust in the UK when attending to children and how these experiences influenced their decision making and provision of care.

## Methods

This original research study has utilised the consolidated criteria for reporting qualitative research (COREQ). This framework was developed to provide a complete checklist for the reports of qualitative studies (Tong et al., 2007) which has been approved by the Enhancing the QUAlity and Transparency Of health Research (EQUATOR) network. This study was initially undertaken as part of a postgraduate academic qualification.

### Study design

With a desire to understand the lived experiences of paramedics and how those experiences made paramedics feel, a phenomenological approach was employed, as it was not desirable to block out similar experiences (Griffiths & Mooney, 2012) or preconceptions experienced by the researcher as a result of his own paramedic background.

### Research team and reflexivity

Qualitative interviews of paramedics to explore their experiences of decision making while managing paediatric emergencies were conducted by a single male researcher who is also the author. Prior to interview, five of the participants were known to the researcher. After expressing interest, each participant received a participant information sheet and consent form detailing the aims of the study and what was asked of them. Before interviews commenced, the researcher outlined his current role and interest in the subject matter and provided the participants with the opportunity to ask questions prior to obtaining their informed consent to take part in the study.

### Sampling

Volunteers from an advertisement in a weekly Trust information bulletin secured an initial sample. Recommendation from volunteer participants to colleagues was responsible for further snowball sampling to provide the remainder of participants. A total of 15 participants were selected and agreed to the interviews, with no withdrawals at any stage.

### Setting

Three interviews were carried out at the participants’ workplace, with the remaining 12 interviews completed remotely. Interviews in the workplace were all held in a private room with only the researcher and the participant present while adhering to social distancing guidelines. For remote interviews, participants and the interviewer chose their own private location at their own discretion.

The 15 participants recruited demonstrated varying degrees of experience and education. All participants are Health and Care Professions Council registered paramedics as detailed in Table 1, which also profiles demographic data. The inclusion and exclusion criteria for participation are demonstrated in Table 2.

**Table 1.** Participant demographics.

Participant	Total length of service (emergency response)	Length of time qualified as a paramedic	Initial qualification gained to achieve registration	Further education in HEI related to paramedicine since qualification	Gender
**1**	More than 2 years	More than 2 years	DipHe	BSc	F
**2**	More than 10 years	More than 2 years	DipHe	None	M
**3**	More than 2 years	More than 2 years	DipHe	None	M
**4**	More than 10 years	More than 10 years	DipHe	BSc	M
**5**	More than 10 years	More than 5 years	DipHe	Currently engaged at BSc level	M
**6**	More than 10 years	More than 2 years	DipHe	BSc	F
**7**	More than 10 years	More than 10 years	BSc	MSc	F
**8**	More than 20 years	More than 20 years	IHCD	DipHe	M
**9**	More than 15 years	More than 2 years	DipHe	None	F
**10**	More than 15 years	More than 2 years	DipHe	None	F
**11**	More than 10 years	More than 2 years	DipHe	BSc	F
**12**	More than 5 years	More than 1 year	DipHe	None	M
**13**	More than 10 years	More than 1 year	DipHe	None	F
**14**	More than 15 years	More than 8 years	DipHe	None	M
**15**	More than 20 years	More than 20 years	IHCD	DipHe BSc MSc	F

BSc = Bachelor of Science; DipHe = Diploma of Higher Education; HEI = higher education institute; IHCD = Institute of Healthcare and Development; MSc = Master of Science.

**Table 2.** Inclusion and exclusion criteria for study sample.

Inclusion criteria	Exclusion criteria
Health and Care Professions Council registered paramedic	Paramedics providing telephone triage only
Attended an emergency call to a patient aged 15 or younger	Clinical decision making by paramedics working in another environment other than the ambulance service
Attended the call within the last month prior to planned interview	

### Data collection

Five questions (Supplementary 1) formed the basis of a semi-structured approach. Salient points noted during recording were utilised as prompts to ask further questions. This provided the opportunity for individual emerging themes to be further expanded upon (Griffiths & Mooney, 2012). This flexible approach is most appropriate when the researcher is also conducting the interviews (Robson & McCartan, 2018). No pilot interviews were completed prior to commencement of the research as a result of time constraints. Audio of all interviews was digitally recorded and transcribed verbatim, with no requirement for repeat interviews. Anonymity was ensured with the allocation of participant codes, as detailed in Table 1.

Interviews had no set time limit, lasting between 18 and 56 minutes, allowing participants to talk freely about their experiences. Accordingly, saturation was not appropriate (Marshall & Long, 2010) due to adoption of a narrative approach. Vigilance by the researcher ensured participants maintained course of the subject matter. There was no re-contact with participants for interview transcript review. Transcription of all interviews was undertaken by a medical secretary who had previous experience of transcribing interviews.

### Analysis and findings

Coding was exclusively conducted by the researcher aside from one interview. A consultant nurse, neutral to the study and with previous qualitative research experience, coded one interview selected at random. Findings between the two data coders in this instance were similar. This approach may not be considered optimum, although Richards (2015) cautions against the reliability of inter-coding.

Data were coded and analysed using thematic analysis. Specifically, the Braun and Clarke (2006) model was employed to search across the dataset to identify patterns. This six-phase approach to analysis (Table 3) is not a linear process progressing through each phase chronologically. Rather, it is recursive, allowing the researcher to move back and forth through the phases as required.

**Table 3.** Braun and Clarke’s six phases of thematic analysis.

**1**	Familiarising yourself with the data
**2**	Generating initial codes
**3**	Searching for themes
**4**	Reviewing themes
**5**	Defining and naming themes
**6**	Producing the report

Conduction of the interviews was the first true stage of familiarisation. Subsequently, listening to the data and repeated reading of the verbatim transcriptions ensured an immersive process. Observations of experiences were listed and documented specific to each participant. This process provided the basis of codes.

Identification of patterns and grouping together commonalities of these codes formed the first basis of themes (Saldana, 2016) which were then reviewed and refined. Discarding of codes due to a lack of repetition contributed to providing a definitive dataset into which themes could then be named. Themes were further assembled into sub-themes, identified by Braun and Wilkinson (2003) as being useful for structure and hierarchy. No software was employed in the coding of the data. The lived experiences of participants presented as a descriptive analysis, supported by anonymised verbatim quotations from the reflective experience, form the final production of the report.

## Results

Five themes were identified from the thematic data analysis of the qualitative interviews:

1. education and training;
2. exposure, experience and confidence;
3. emotion and social circumstances;
4. equipment and protocols; and
5. provision of care.

### Education and training

Education and training featured heavily in responses from all participants and produced three sub-themes: (1) registrant level of education and training; (2) mandatory training; and (3) continuing professional development (CPD).

#### Registrant level of education and training

Although registrant level of education was variable between participants, education central to paediatrics was considered to be lacking:

GM5: *Through the paramedic course I don’t feel we got trained enough on the paediatric side. I think it was just aimed more at adults […] we need a little bit more training I think.*

GM1: *I don’t think we have been given the training and the tools to fully assess a child.*

Education primarily prepared participants for attending to paediatric patients presenting in peri-arrest or cardiac/respiratory arrests. There was little training for low acuity ailments or conditions more common to paediatric patients:

C&M4: *Most of the training I received around paediatrics was primarily around cardiac arrest and resus. We weren’t really educated much in the assessment of minor injuries and illnesses. Most of the scenarios were all geared around critically ill kids.*

GM4: *My recollection of university really was pre-dominantly, with paediatrics, was just about paediatric BLS and ALS. There may only have been an afternoon session or two about paediatric illness. Certainly never anything about how to deal with families; and how to talk with children.*

Complementary practice placements are common practice within paramedic education; experiences were largely negative:

GM4: *Awful […] In terms of accommodating our job role and our learning needs it was two days with not much real structure.*

GM6: *I was told at the beginning do not approach paediatric theatre. He [the anaesthetist] doesn’t want you in the room, he doesn’t want to see you.*

#### Mandatory training

Discussion of mandatory training experience broadly reflected the experience of registrant level of education for most participants:

C&M4: *It was more resus and kind of, maybe, airway management of kids, it was nothing to do with things that weren’t critical. The kind of injuries or illness that parents worry about, rashes and weird things like that, you don’t really get much insight to that kind of thing really.*

#### Continuing professional development

The importance of CPD as a Health and Care Professions Council (2018b) registrant requirement was recognised:

GM7: *I didn’t realise how much I’d forgotten until I done something like that [CPD].*

GM3: *I try and keep up with stuff, I’m on Spotting the Sick Child and Two Paeds in a Pod. I try to keep up to date with things.*

GM3: *It’s very much up to you to go and keep yourself current.*

### Exposure, experience and confidence

Confidence (or lack of) appears to stem from experience for most. This experience can be gained from exposure in a professional and personal capacity:

C&L3: *I think confidence can be underpinned with frequency of exposure […] If you’re assessing paediatric cases every day I think you become more proficient.*

GM2: *I’ve never done that, I wouldn’t be confident cannulating a toddler. I’ve never actually cannulated a child to be fair.*

This lack of confidence in performing interventions led GM3 to comment on the impact this could have on quality of care:

*Would you want Ibuprofen if a car had just run over your leg? Seriously? […] People are scared of cannulating kids or trying to […] I think we are poor at managing children’s pain.*

The impact of differing family dynamics is highlighted in these observations of two participants:

C&M4: *Kids can be quite scary in this job; if you haven’t got kids yourself, it’s a bit of an unknown quantity.*

C&M5: *As learning experiences, both of my kids became poorly which directly influenced how I managed my patients.*

### Emotion and social circumstances

Participants discussed the impact of their emotions on their decision making. The term ‘gut feeling’ was prevalent in decision making:

GM2: *Gut feeling and I would rather err on the side of caution.*

C&M5: *It’s that subconscious gut feeling, particularly in paeds, that I find is a massive driver in decision making as much as your pathways are.*

Nervousness and risk also drove decision making:

C&M3: *With children you are always a bit more nervous and a bit more erring on the side of caution.*

C&L3: *To sum it up, my appetite for risk when dealing with and treating a child is an awful lot lower than my appetite for risk when dealing with adults.*

The phrase ‘gut feeling’ was observed from the family perspective also:

GM2: *You don’t want them to panic just because you are there.*

C&L3: *I take into consideration the parents’ feelings.*

C&M1: *If they are saying something is not right then you should probably trust what they are saying.*

### Equipment and protocols

The Joint Royal Colleges Ambulance Liaison Committee produces national clinical practice guidelines for paramedics which are based on best available evidence. These guidelines are mainly in reference to acute, severe and life-threatening emergencies:

C&L1: *For example an allergic reaction or cardiac arrest, I would definitely be checking the drug dosages and things like that to make sure I was prepared.*

Local procedures such as the application of the Manchester triage system (MTS) have been developed. The MTS is a tool for clinicians to use to aid decision making. GM2 commented:

*So for medical under two years we have to take them to ED […] It has its advantages and disadvantages and on occasion you are glad it is in place because you don’t have to commit [to making a decision].*

While GM3 acknowledged the restrictions of guidelines, her opinion on the application of them differed:

*We are restricted by MTS if you follow it to the age limit […] if I am honest I don’t always follow it if I think there is a more sensible route I will follow what I think. I’ll never just leave at home […] I would always refer on.*

The ability to perform clinical assessments of children was a topic discussed by many, with C&M1 highlighting advantages for clinicians outside of the ambulance service:

*I think they can do a bit more than us like looking in ears, back of the throats properly, stuff like that.*

### Provision of care

Following clinical assessment, a decision surrounding provision of care is required. For many there was an expectation, from families, that an ambulance attendance would result in transport of patients to hospital:

C&M4: *I think parents feel like they want to go to hospital […] they want definitive answers from definitive people. They may not trust the word of an ambulance man.*

C&M2: *I feel that the general public perception is, if we turn up for their child they expect to go to hospital.*

Ambulance culture may have contributed to this expectation:

C&L1: *I feel that there is a culture that we will err on the side of caution; historically all children have gone to A&E.*

Alternatives to hospital are available, but C&L2 acknowledged that for referral to community services, you had to be confident:

*You have to be really in confident in that decision […] to discuss an alternative treatment pathway.*

When this confidence was present, clinicians experienced a mixed but largely negative experience:

C&L3: *As regards referrals with kids I have found that other primary carers, GPs, are very risk averse […] extremely risk averse. I think it’s pretty much always been a conveyance outcome.*

GM7 highlighted that this experience was related to the provider:

*We’ve got two out-of-hours providers. M is compliant and as brilliant as you’d ever want. G it’s like banging your head against a brick wall. It’s a postcode lottery.*

Leaving a patient at home was a less popular option:

C&M1: *I think it’s quite hard to leave any child at home especially with the rules around the [under] two years [old]. […] I, because of his age, wouldn’t feel comfortable leaving him at home.*

GM5 agreed with C&M1 but acknowledged it had a potentially damaging impact:

*You could also just give the Mum and Dad the ownership back to keep an eye on their own child and I think we take that away from them a lot. That makes the parents question their ability as to what they can manage.*

## Discussion

With the study of paramedic practice and its evolvement being in its infancy (Givati et al., 2018), the search for evidence will provide a current challenge for researchers. Registrant level of education has developed dramatically (College of Paramedics, 2019), transitioning from in-house training of Institute of Healthcare and Development qualification to higher education institutes (HEIs). All participants were in agreement of the importance of paramedic education featuring resuscitation and life-saving treatments. Education for paramedics is primarily focused on this form of emergency medicine (O’Meara et al., 2017). For participants, this was a small portion of actual work. In addition to this necessary training, a desire for specific paediatric education more reflective of primary care presentations and age specific was expressed.

Brady (2018) discusses confidence, noting there is little research available on paramedic confidence in clinical practice. Lack of exposure and confidence were found to be directly linked during focus groups from Brady’s (2018) research. Experience was a key factor for many paramedics. A link between experience and confidence, which O’Connor and Leonard (2014) recognise as an important factor when working in child health and social care, was evident. Participants observed that confidence could impact on quality of care. Various authors (Hayes, 2003; McIntosh-Scott et al., 2013) have also commented on this link between confidence and patient outcome.

Exposure in a professional capacity is difficult to control as paramedics generally do not have control over which incidents they will respond to. Exposure in a personal capacity will be dependent on family dynamics, although parenthood itself should not be considered as the only exposure to children as extended families may also contribute.

Roland and Matheson (2012) found that clinicians with the necessary experience in assessing children do not tend to rely on guidelines or tests to make decisions. Conversely, all participants of this study relied on guidelines for severely unwell children and those in cardiac arrest regardless of experience. Decision tools in paediatrics are seldom as sensitive and specific as we would desire (Roland & Snelson, 2019). Accuracy of gut feeling in clinical decision making is variable but shows potential for identifying sick children when compared with individual clinical features (Van den Bruel et al., 2010). Gut feeling of both clinicians and family members featured as a factor when considering emotion.

Paramedics with additional training displayed improved rates of non-conveyance (O’Cathain et al., 2018). Participants who engaged more in additional training were more likely to refer to community services. The ability to perform referral was impacted by availability and suitability within the area and the constrictions of guidelines. A desire to err on the side of caution was observed.

### Limitations

This study is limited as the data were taken from one ambulance service and as such cannot reflect the experiences of all paramedics, although literature does support the themes identified. Regardless, the aim was to explore lived experiences and as such the findings of the study are presented for the reader.

## Conclusions

Education and training are critical factors in decision making. As well as training for life-threatening emergencies, broadening of paediatric education would be welcome. The Health and Care Professions Council (2018a) has raised the threshold for entry to the register from certificate to a full honours degree from 1 September 2021. HEIs, in conjunction with the College of Paramedics, could take advantage of an opportunity to introduce a paediatric-specific module with a focus lent to subject areas such as communication and lower acuity, paediatric-specific conditions. To complement additional education, adoption or development of national guidelines for lower acuity complaints would further equip paramedics with a robust process for decision making. When developing pathways and education, consideration should be given to colleagues who are younger or less experienced.

Ambulance services should investigate the prospect of developing paediatric specialist practitioners within their workforce. Creation of specialists who primarily respond to paediatric patients should contribute to negate factors identified surrounding exposure, experience and confidence. It is acknowledged that this would not be a wholesale resolution for all paramedics. Support from a specialist workforce for education and decision making would further equip Trusts to empower the wider workforce. Development of a national network of paediatric specialists within UK ambulance services could enable Trusts to support each other in development, and in sharing and in learning and resources.

Improved patient experience as patients access the most appropriate level of care would be in line with the vision to make every contact count (Health Education England, 2020). Community services and parental empowerment will be integral to any future success and will possibly be more difficult to influence. ED attendances for children since the start of the COVID-19 pandemic were down approximately 30% and increasing (Isba et al., 2020). COVID-19 witnessed a change in approach to healthcare. Early lessons learnt may provide the opportunity to capitalise on this change safely.

The ‘long term plan’ (NHS England, 2019) recognises the importance of research and innovation for medical advancement. Considering the paucity of literature in paramedicine, never has a statement felt more apt. Further research focused on specific areas of pre-hospital paediatric healthcare and paramedic experience would enhance understanding of paramedic behaviour and provide an evidence base to drive innovation forward. Service evaluation of the impact of specialist paediatric roles could provide a template for replication for other specialties such as frailty, maternity or mental health.

## Acknowledgements

The author would like to thank the participants who gave up their time in the midst of a worldwide pandemic to take part in interviews, Mr Matt Holland (Library & Knowledge Services for NHS Ambulance Services in England) for his support with research for this project and Sandra Igbodo (North West Ambulance Service research manager) for her support and confidence in Jeff Hetherington.

## Author contributions

JH is the lead researcher involved in this study. IJ contributed to the conception and design of the study and undertook a critical review of the final manuscript. JH acts as guarantor for this article.

## Conflict of interest

None declared.

## Ethics

Participants received information sheets pertaining to the study and involvement prior to interviews being undertaken. Approval was received from participants at the beginning of the interviews. Organisational approval for the study was achieved via the research team. Ethical approval for the study was received from Liverpool John Moores University (LJMU) ethics committee.

## Funding

None.
